# Bluetooth Noise-Canceling Headphones Improve the Quality of Ophthalmic Exams in Patients With Hearing Loss: A Randomized Controlled Trial

**DOI:** 10.7759/cureus.60090

**Published:** 2024-05-11

**Authors:** Benjamin M Glanzer, Malik Ladki, Metha R Chea, Lena Hummel, Brian McKinnon, Biai Dominique E Digbeu, Kevin H Merkley, Atish Amin, Praveena K Gupta

**Affiliations:** 1 Ophthalmology, University of Texas Medical Branch at Galveston, Galveston, USA; 2 Otolaryngology - Head & Neck Surgery, University of Texas Medical Branch at Galveston, Galveston, USA; 3 Biostatics, University of Texas Medical Branch at Galveston, Galveston, USA; 4 Ophthalmology and Visual Sciences, University of Texas Medical Branch at Galveston, Galveston, USA

**Keywords:** elderly, ophthalmic exam, communication, headphones, hearing-loss

## Abstract

Introduction: This study tests the utilization of Bluetooth noise-canceling headphones in improving the quality of eye exams in patients with hearing loss. This prospective study was approved on ethical standards by the University of Texas Medical Branch (UTMB) Institutional Review Board (Approval No. 22-0079) and registered with the National Institutes of Health (NCT05420038).

Methods: UTMB patients above 55 years of age were screened for hearing loss using soundcheck audiometry. Twenty-nine subjects answered pre-recorded ophthalmic exam questions that solicited precise responses. As controls, subjects were randomly administered half of the questions via headphones and half via a smartphone at normal speech volume (60 decibels). Points were awarded for responses demonstrating comprehension, and a post-exam survey was collected.

Results: Collectively, the mean score was 1.79 with headphones versus 0.96 with control on the Amsler grid segment and 1.90 with headphones versus 0.97 with control on education questions (p=0.001). Between red zone and yellow zone hearing loss patients, the more severe red zone group answered significantly better in both Amsler (1.78 versus 0.50; p=0.0003) and education questions (1.88 versus 0.44; p<0.0001) with headphones. The yellow zone group answered better with headphones overall but failed to reach significance. Post-exam survey indicated that 28 of 29 patients (97%) preferred the headphones during ophthalmic exams.

Conclusion: Patients with hearing loss demonstrated better comprehension with Bluetooth headphones. These low-cost devices show great promise at improving effective, compassionate communication between providers and hearing loss patients.

## Introduction

Hearing loss is a highly prevalent cause of disability among older American adults, affecting up to half of individuals aged 60 and above, and two-thirds of those older than 70 years of age [1]. This age-related condition impacts numerous aspects of the affected individuals’ lives and has been associated with lower quality of life, depression, social isolation, and functional decline [2-5]. In healthcare settings, hearing impairment poses a frequent barrier to communication in aging patients. Effective communication between providers and their patients is critical in delivering high-quality care and building a strong relationship based on compassion and respect [6].

The use of hearing assistance devices has been shown to mitigate many of the adverse outcomes associated with hearing loss. For example, the use of cochlear implants or hearing aids has been shown to significantly improve cognitive, social, and behavioral domains in the lives of those affected [7,8].

Although hearing aids are widely available, it has been documented that many individuals with moderate to severe hearing loss fail to use these tools [9]. Common barriers to using hearing aids include high costs, the need for frequent repairs, and issues with whistling and feedback. Amongst individuals who do possess hearing aids, data has been collected that shows that anywhere from 3% to 24% do not use them because of physical discomfort, difficulty of use, or social perceptions [10]. Additionally, the use of personal protective equipment such as facemasks in a clinic or hospital setting can exacerbate verbal communication issues for individuals who have come to depend on lip-reading to supplement their hearing deficiency [11]. Therefore, although hearing aids have been available for years, their use has not addressed the issue of ineffective communication in clinics for patients with hearing loss.

The fastest-growing cohort of individuals in the United States is those aged 85 and older, which is predicted to rise from around six million in 2014 to around 19 million by 2050 [12]. As the baby boomer generation ages, the number of elderly patients needing health care in areas of the eye and ear will increase in addition to other health issues. Many would agree that communication with elderly, especially with those that have age-associated hearing loss, can often lead to frustration, loss of effective communication, and anxiety between various levels of family members and care providers. In a hospital setting, poor hearing can lead to misunderstanding of treatment and resulting noncompliance. Therefore, there is a growing need to find an effective way to communicate with these patients, especially in a health clinic setting.

Currently, amplification devices that boost volume have been shown to be effective in enhancing communication with older patients with hearing loss in nursing homes, hospitals, and palliative care units [13]. These devices are either sparsely used or do not have a noise-canceling ability. Bluetooth noise-canceling headphones are cost-effective and user-friendly devices that can be readily deployed in a similar manner due to their amplification capacity and ability to reduce unwanted background noise. However, there is a lack of studies evaluating the use of Bluetooth noise-canceling headphones in a clinical setting.

This study aims to assess the use of Bluetooth noise-canceling headphones in delivering information to patients who have moderate to severe hearing loss during ophthalmic exams. As the population ages, more elderly individuals with hearing impairment will engage with eye care providers. Innovations like such may enable patients to understand the provider’s questions, reduce appointment time, and improve patient-physician communication.

This article was previously posted to the Preprints.org preprint server on October 18, 2023.

## Materials and methods

This prospective study was approved on ethical standards by the University of Texas Medical Branch (UTMB) Institutional Review Board (IRB Approval No. 22-0079). This study design followed the guidelines as per the Consolidated Standards of Reporting Trials (CONSORT) 2010 statement. The research protocol, data collection sheet, post-study survey, and consent forms were all approved under the expedited review procedure 45 CFR 46.110 under the code of federal regulations (CFR). This study is Health Insurance Portability and Accountability Act (HIPAA) compliant and has been registered with the National Institutes of Health (NIH) (NCT05420038) available at https://clinicaltrials.gov/ct2/show/NCT05420038. All participants provided written informed consent for inclusion in the study, collection of data, and publication prior to enrollment in the study.

Subjects were individuals 55 years of age or older seen at UTMB ophthalmology and otolaryngology clinics. Individuals suspected of moderate to severe hearing impairment were checked with the Soundcheck Hearing Test phone application (version 2.0.6, Starkey Hearing Technologies, Eden Prairie, USA) to confirm the possible hearing loss. Only patients that fit those criteria were enrolled in the study. Individuals with a history of corrective procedures performed for hearing loss, cognitive impairment, or who are part of a vulnerable population were excluded from the study.

Patients in this study were first screened with the Soundcheck hearing test that was administered through a smartphone connected to Anker Soundcore Life Q20 Bluetooth noise-canceling headphones (Anker Innovations, Shenzhen, China). Soundcheck is a basic true-tone hearing loss screening tool that measures patient hearing thresholds at 500, 1000, 2000, and 4000 hertz. Hearing grade on this app is based on the World Health Organization (WHO) hearing loss guidelines [14]. Patients took the Soundcheck hearing test on a smartphone device with Bluetooth headphones, and their results were either “normal hearing” (green zone and better) or “possible hearing loss” (yellow zone and red zone, moderate and severe hearing loss respectively). If patients wore hearing aids, they were instructed to remove them for the duration of the study. Only patients who scored in the red zone (120 dB threshold) or yellow zone (90 dB threshold) bilaterally were included in the study. Once hearing loss was established, patients received two sets of questions including an Amsler grid to be held at arm's length, and they were asked to cover one eye while answering the questions and they also received patient education questionnaires. An online random sequence generator was used to generate a sequence of four numbers to determine the order of questions.

Amsler grid questions

Question 1: Do the lines appear wavy or crooked? If so, please say out loud “the lines appear wavy”. If not, please say “the lines do not appear wavy.”

Question 2: Do the boxes appear the same size? If so, please say out loud “the boxes appear the same size”. If not, please say “the boxes appear different sizes.”

Question 3: Are there any holes or dark areas in the grid? If so, please say out loud “there are dark areas in the grid.” If not, please say “there are no holes or dark areas.”

Question 4: Can you see all four corners of the grid while focused on the center dot? If so, please say out loud “I can see all four corners”. If not, please say out loud “I cannot see all four corners.”

**Figure 1:**
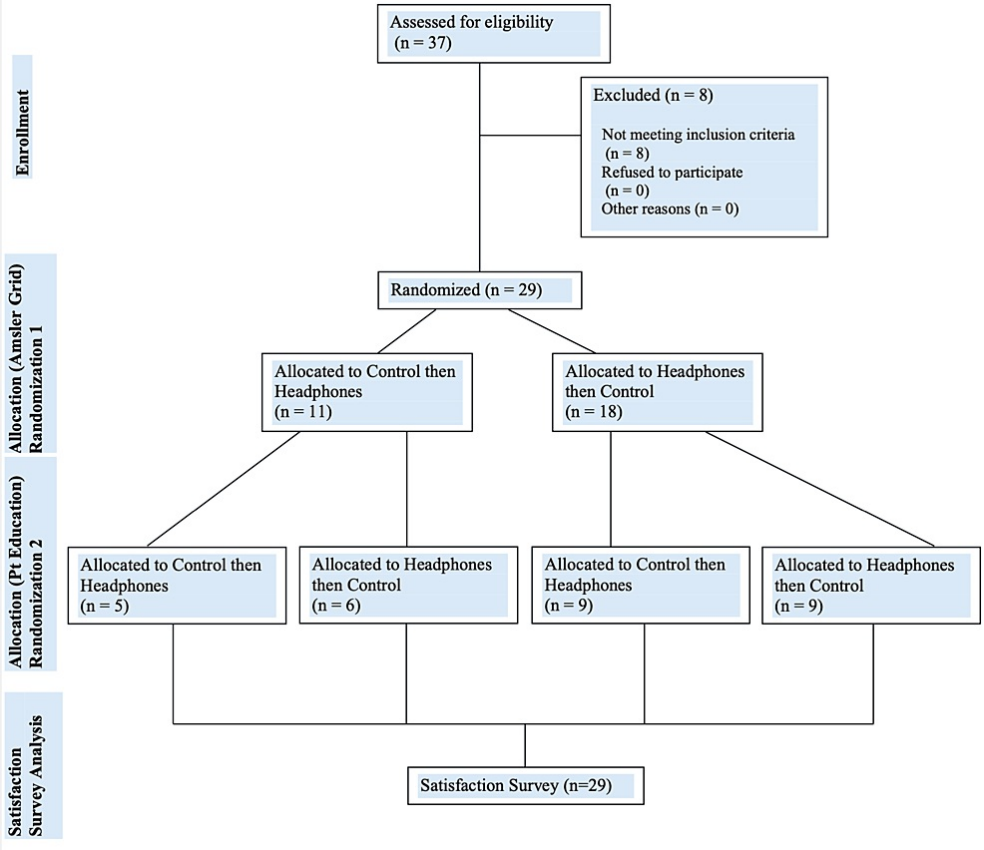
Patient randomization flowchart demonstrating the order in which subjects completed the various components of the study to prevent learning bias

In addition to receiving randomized questions, the order in which patients received either the intervention or the control was randomized. Some patients received the first two randomly selected questions via the control, a smartphone held at two arm’s lengths from patients at the phone’s mid-level volume. Other patients received the first two randomly selected questions via Bluetooth noise-canceling headphones. After the first two questions, patients would receive the third and fourth questions via the alternate modality, either intervention or control. The patient randomization flowchart illustrating the recruitment of subjects and the double rounds of randomization to the order of the headphones intervention and the control is shown in Figure 1.

If the patient answered correctly, they received a score of 2. If they responded with any phrase other than the potential answer choices, but in a way that demonstrated partial hearing and comprehension, they received a score of 1. If they did not hear the question or asked for repetition, they received a score of 0. After the Amsler grid questions were completed, four patient education questions were asked and scored with the same methods.

Patient education questions

Question 1: “Whenever patients complain of dry eyes, we tell them to use the brands Systane or Refresh four times per day. Can you tell us how many times a day to use Systane or Refresh?”

Question 2: “Blepharitis is a condition that occurs when small oil glands in the inner eyelid become inflamed. Can you please repeat what you just learned about Blepharitis?”

Question 3: “Allergic conjunctivitis can cause redness and itchiness in the eye. To treat it, patients are encouraged to use Pataday drops once per day. How many times per day should patients use Pataday?”

Question 4: “Glaucoma is a condition that can occur when excess pressure builds up in the eye because of drainage problems. Can you please repeat back what you just learned about glaucoma?”

Data was collected on the use of Bluetooth headphones (right or left eye), order of Amsler grid questions, order of patient education questions, scores on Amsler grid questions with and without headphones (0-2), scores on patient education questions with and without headphones (0-2).

Statistical analysis was conducted with the assistance of the UTMB biostatistics department with a pre-decided level of significance of p<0.05. All data were collected, combined, and analyzed using descriptive statistics, Cohen's K coefficient, t-test, Fisher's exact test, Chi-squared test, and prediction models for significance.

## Results

Over the course of our study, we approached 37 patients of UTMB ophthalmology and audiology clinics aged 55 or above for inclusion in the study. Eight of those 37 individuals were excluded after screening with the Soundcheck hearing test due to normal hearing or minimal hearing loss. Table 1 shows the demographic data of the subjects recruited in the study including age, sex, race, whether or not they wear hearing aids, and their Soundcheck hearing grade.

**Table 1:** The demographic data of the subjects recruited in the study including age, sex, race, whether or not they wear hearing aids, and their Soundcheck hearing grade.

	Participant demographics	Participants (n=29)
Age (Years)		
	Mean (SD)	77.2 (8.36)
	Median [Min, Max]	76 [64.0, 96.0]
Sex		
	Female	10 (34.5%)
	Male	19 (65.5%)
Race		
	White	24 (82.8%)
	Black	2 (6.89%)
	Hispanic	2 (6.89%)
	Asian	1 (3.45%)
Wears Hearing Aids?		
	Yes	15 (51.7%)
	No	14 (48.3%)
Hearing Loss		
	Yellow Zone (Moderate)	11 (38%)
	Red Zone (Severe)	18 (62%)

The included subjects had a mean age of 77.2 ± 8 years and were mostly male (n=19) (65.5%). The majority of the sample’s race was White (n=24) (82.8%) followed by (n=2) (6.89%) Black, (n=2) (6.89%) Hispanic, and (n=1) (3.45%) Asian. About half of the sample wore hearing aids regularly (n=15) (51.7%). Our screening placed 18 subjects in the severe hearing loss zone (n=18) (62%) and 11 in the moderate hearing loss zone (n=11) (38%). Of the total included sample, 18 subjects were randomized to begin the Amsler grid segment of the study with the Bluetooth headphones and 11 were randomized to begin with the smartphone control, after which they each used the alternative. Following this, the first group of 18 was randomized again to complete the patient education segment of the study with nine subjects (n=9) (50%) using the headphones and nine subjects using the control (n=9) (50%), after which they each used the alternative. The n = 11 group was similarly randomized with six subjects (n=6) (54.5%) using the headphones first and five (n=5) (45.5%) using the control.

According to the paired t-test, the headphone intervention resulted in more questions answered correctly compared to the non-headphone controls in both the Amsler grid and patient education sections (P=0.0011 and P<0.0001 respectively). Table 2 shows the number of questions answered correctly with headphones and without headphones among all patients.

**Table 2:** Overall headphone intervention results reporting the mean number of correct answers given with headphones versus without, as well as the mean, standard deviation (SD), and P-value (<0.05). P-value was determined to be significant at P<0.05.

	Mean number answered correctly				Difference	
Question type	N	With headphones	Without headphones	Mean (SD)		P-value
Amsler	29 (100%)	1.79 (0.62)	0.96 (0.98)	0.83 (1.23)		0.0011
Education	29 (100%)	1.90 (0.41)	0.97 (0.98)	0.93 (1.03)		<0.0001

**Figure 2:**
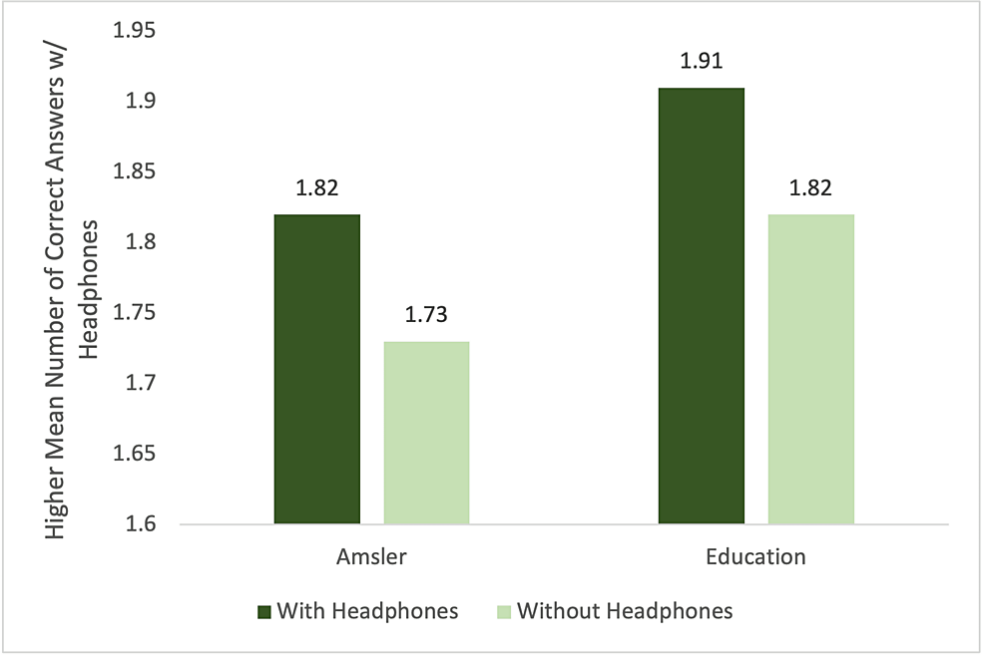
Yellow zone (moderate hearing loss) Soundcheck group performance by mean number of correct answers given with headphones compared to without.

In the patients with yellow zone (moderate hearing loss) group, both the Amsler grid exam and education segment demonstrated a higher score with the headphones but failed to reach significance (1.82 versus 1.73; P=0.76 and 1.91 versus 1.82; P=0.68, respectively). Figure 2 shows the graph for the performance of subjects with moderate hearing loss group.

**Figure 3:**
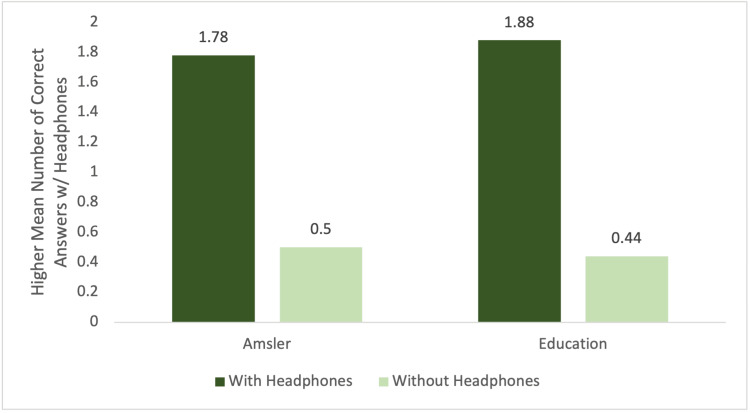
Red zone (severe hearing loss) Soundcheck group performance by mean number of correct answers given with headphones compared to without.

In the patients' red zone (severe hearing loss) group, there was statistical significance in the Amsler grid exam (1.78 versus 0.50; p=0.0003) and patient education segment (1.88 versus 0.44; P<0.0001). Figure 3 shows the graph for the performance of subjects in the red zone (severe hearing loss) group.

**Figure 4:**
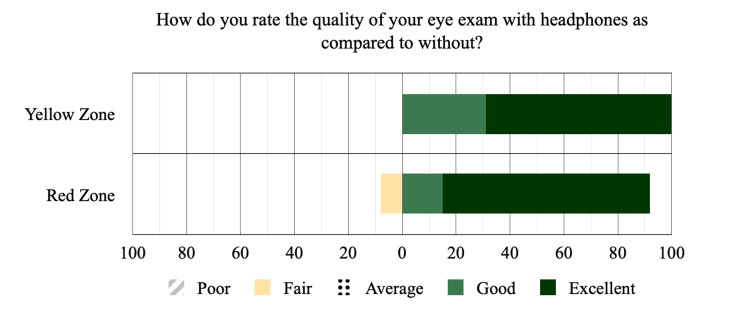
Satisfaction survey bar graph displaying subject's survey results as percentage totals per Soundcheck hearing grade category. Yellow zone represents moderate hearing loss. Red zone represents severe hearing loss.

Post-exam survey indicated that (n=28) 97% of patients preferred the use of headphones across both groups despite differing degrees of performance improvement, with (n=14) 77.78% of red zone subjects rating it “excellent”, (n=3) 16.67% rating it “good” and (n=1) 5.56% rating it as “fair”. Of the yellow zone subjects, (n=7) 63.64% rated the use of headphones as “excellent” and (n=4) 36.36% rating it “good”. Figure 4 shows the satisfaction survey bar graph displaying subjects' survey results as percentage totals per Soundcheck hearing grade category. Recurring feedback from those who preferred the use of headphones included ease of listening, less ambient background noise, and better comprehension regardless of actual differences in performance.

## Discussion

In this clinical trial among 55 and older ophthalmology and audiology patients with hearing loss, we found that supplementing patients with Bluetooth noise-canceling headphones significantly increased comprehension and patient satisfaction. Hearing deficits documented using Soundcheck true-tone audiometry allowed us to objectively assess subjects’ hearing and thus more precisely isolate hearing loss as a variable. Placing subjects into two groups (yellow and red zone) based on severity further narrowed this focus. Of note, the use of hearing aids among these patients was only (n=15) 51.7% despite detectable deficits, aligning with literature findings of low hearing aid usage in the United States among this population [10].

Question design was intentionally crafted so that subjects must speak highly specific responses in order to receive credit for full comprehension, thus limiting bias in data collection. Furthermore, the pre-recording of questions allowed for standardization of a single voice’s wording, tone, volume, accent, and clarity. The removal of hearing aids prior to the study eliminated their usage as a confounding variable. Allowing subjects to serve as their own controls eliminated the need for different control and intervention groups, thus limiting potential confounding biases between the two. Questions were not repeated to subjects in order to reduce learning, and the randomization of questions reduced potential bias between questions. We prevented additional bias from arising as patients progressed through the study by adding two rounds of randomization, scrambling the orders in which patients were given the intervention and control per segment. Amsler grid exam and patient education were used to simulate a routine clinical encounter.

Analysis showed a substantial jump in comprehension using the Bluetooth headphone intervention. Importantly, these gains are almost entirely within the severe red zone group according to the analysis of statistical significance. Among this group, the differences in comprehension with headphones were remarkable and showed great promise for boosting patient understanding. Because of the differences in results between yellow and red zone groups, our study determined that any objective comprehension benefits of the intervention are dependent on the degree of hearing loss. Interestingly, despite their large differences in comprehension gains, both groups overwhelmingly preferred the use of headphones according to the post-exam survey. Their responses suggest that while patients with less severe hearing loss may be physically capable of overcoming their hearing deficits, the use of Bluetooth headphones makes this much less of a challenge and leads to gains in an equally important variable of patient comfort and satisfaction.

In comparison to similar studies, the current study is able to establish overall patient satisfaction with the implementation of an assistive hearing device similar to the use of personal amplifiers as seen in other studies. While other studies focused on measures of improvement in hearing with assistive devices regarding general instructions about discharge or medications which can vary in complexity on a patient-patient basis, this current study aims to help support how nuanced and patient-specific information about a patient’s specific diagnosis, prognosis, or instructions related to a specialty-specific clinic may be better communicated with the patient. This is important since the level of specificity and the level of personalized care plans might present differently for a patient in a clinical ophthalmology setting, emergency department setting, or an oncology clinic [13].

One of the limitations of this study is the sample size, which may limit the power of this study. However, the number of patients included served as their own controls that enabled statistically significant results for the primary and secondary outcomes. Additionally, the use of a smartphone is not an exact replication of or substitute for human voice. However, the same audio recording was used for higher accuracy of the findings. Further, Soundcheck’s audiometry, although rapid and useful as a screening tool, cannot replace the gold standard Soundbooth audiogram administered by an audiologist. While the authors acknowledge that understanding speech is an engaging process that requires intact cognitive function, the current study did not test the cognition condition of the patients [13,15]. However, patients with a documented history of severe cognition loss were excluded from the study. The important next steps include the expansion of this study to larger samples of aging populations and the development of a smartphone-based app that instantaneously delivers high-fidelity speech audio from a clinician’s device to Bluetooth noise-canceling headphones that may be affordably used in virtually any clinic environment.

## Conclusions

Age-related hearing loss is a common issue among the aging population that poses a range of threats, including uncomfortable patient experiences, difficult communication, and misunderstanding of instructions with resulting noncompliance. While this is a familiar, frustrating dilemma, it is frequently overlooked in hospital settings, thus leading to compromised patient care. We report a novel, user-friendly solution to this preventable scenario through the deployment of Bluetooth noise-canceling headphones in ophthalmology clinics and in other departments that provide care to high volumes of aging patients with hearing loss. They have become low-cost, high-quality listening devices that can provide undisturbed audio amplification and background noise reduction to elderly patients with hearing loss. These devices show great promise in improving effective, compassionate communication between providers and hearing-loss patients. As reported in our study, simple innovations and deployment of the existing low-cost technology of Bluetooth headphones could provide an immediate and significant boost to the quality of care received by aging patients with hearing loss.
